# Functional and Morphological Correlates in the Drosophila LRRK2 *loss-of-function* Model of Parkinson’s Disease: Drug Effects of *Withania somnifera* (Dunal) Administration

**DOI:** 10.1371/journal.pone.0146140

**Published:** 2016-01-04

**Authors:** Francescaelena De Rose, Roberto Marotta, Simone Poddighe, Giuseppe Talani, Tiziano Catelani, Maria Dolores Setzu, Paolo Solla, Francesco Marrosu, Enrico Sanna, Sanjay Kasture, Elio Acquas, Anna Liscia

**Affiliations:** 1 Department of Life and Environmental Sciences, University of Cagliari, Cagliari, Italy; 2 Nanochemistry Department, Fondazione Istituto Italiano di Tecnologia, Genova, Italy; 3 Department of Biomedical Sciences, University of Cagliari, Cagliari, Italy; 4 Institute of Neuroscience, National Research Council (CNR), Monserrato, Cagliari, Italy; 5 Department of Public Health, Clinical and Molecular Medicine, University of Cagliari, Cagliari, Italy; 6 Pinnacle Biomedical Research Institute, Bhopal, India; INSERM / CNRS, FRANCE

## Abstract

The common fruit fly *Drosophila melanogaster* (*Dm*) is a simple animal species that contributed significantly to the development of neurobiology whose leucine-rich repeat kinase 2 mutants (LRRK2) *loss-of-function* in the WD40 domain represent a very interesting tool to look into physiopathology of Parkinson’s disease (PD). Accordingly, LRRK2 *Dm* have also the potential to contribute to reveal innovative therapeutic approaches to its treatment. *Withania somnifera* Dunal, a plant that grows spontaneously also in Mediterranean regions, is known in folk medicine for its anti-inflammatory and protective properties against neurodegeneration. The aim of this study was to evaluate the neuroprotective effects of its standardized root methanolic extract (*Wse*) on the LRRK2 *loss-of-function Dm* model of PD. To this end mutant and wild type (WT) flies were administered *Wse*, through diet, at different concentrations as larvae and adults (L^+^/A^+^) or as adults (L^-^/A^+^) only. LRRK2 mutants have a significantly reduced lifespan and compromised motor function and mitochondrial morphology compared to WT flies 1% *Wse*-enriched diet, administered to *Dm* LRRK2 as L^-^/A^+^and improved a) locomotor activity b) muscle electrophysiological response to stimuli and also c) protected against mitochondria degeneration. In contrast, the administration of *Wse* to *Dm* LRRK2 as L^+^/A^+^, no matter at which concentration, worsened lifespan and determined the appearance of increased endosomal activity in the thoracic ganglia. These results, while confirming that the LRRK2 *loss-of-function* in the WD40 domain represents a valid model of PD, reveal that under appropriate concentrations *Wse* can be usefully employed to counteract some deficits associated with the disease. However, a careful assessment of the risks, likely related to the impaired endosomal activity, is required.

## Introduction

Parkinson’s disease (PD) is the second most common neurodegenerative disorder[1] affecting 2% of the population over 60 years with an increasing incidence over age 85 [2]. The progressive loss of dopaminergic neurons in the substantia nigra of the midbrain leads to a deficiency of dopamine causing the typical motor symptoms such as tremor, bradykinesia and rigidity [3][4]. Although the etiopathogenesis is not fully understood and most cases seem sporadic, genetic variables play a key role in the predisposition to PD onset with at least 5 to 10% of PD patients clearly associated with genetic factors[5]. Indeed, since the seminal paper of Polymeropoulos et al. [6], which identified the first mutation related to PD in the alpha-synuclein gene, other genes involved in the etiology of familial forms of parkinsonism have been discovered[7–15]. Among them, the identification of several leucine-rich repeat kinase 2 (LRRK2) gene mutations has opened a novel scenario in Parkinson’s disease genetics[16]. In fact, the G2019S LRRK2 mutation is the most common in Caucasian patients occurring in 1–2% of sporadic cases of PD [17][18], while other mutations, such as the G2385R variants contribute to the susceptibility to develop PD especially in Chinese patients[19]. LRRK2 encodes for a protein with a number of independent domains that is expressed, although at a low level, in all tissues. In the brain it is found in the cortex, striatum, hippocampus, cerebellum, and at the level of the dopaminergic neurons in the substantia nigra [20–23]. Most mutations of this gene are associated with a late onset Parkinsonism [15]. Mutations of the gene LRRK2 that elicit the disease occur at the level of the functional domain Roc (R1441C and G), at the level of the COR (Y1699C and R1628P) and of MAPKKK domains (G2019S and I2020T) and in only one of the WD40 domains (G2385R)[11][15][24]. This latter is known to be crucial in several basic cell functions such as vesicle sorting during endocytosis and exocytosis of synaptic vesicles as well as vesicle-mediated transport and cytoskeleton assembly [25][26]. The role of the WD40 domain is suggested to be crucial in controlling the LRRK2-regulated kinase activity having a critical role in the self-interaction and autophosphorylation-mediated mechanisms of neuronal toxicity [27]. Accordingly, deletion of this domain has been shown *in-vitro* to cause the reduction of the kinase activity that is restored over-expressing the gain of function mutation of the gene[28].

Translational animal models are particularly useful in studying neuronal dysfunction and investigating the etiology and molecular aspects of neurodegenerative diseases. Among the animal species that significantly contributed to the development of these studies, the *Drosophila melanogaster* (*Dm*) represents a simple, yet experimentally and translationally powerful, organism that contributed significantly not only to the development of neurobiology but also to the progress of knowledge on neurodegenerative diseases. Notably, most of the genes implicated in familial forms of PD have a counterpart in this insect [29], and *Dm* mutants of PD have been genetically engineered to model key features of the human condition and have been successfully used in studying PD pathogenesis and in exploring new strategies of disease treatment [30–33]. Previous studies on LRRK2 PD form using *Dm* mutants (dLRRK2) did not clarify the role of LRRK2 in Drosophila, both in mutants *gain-of-function* for the kinase domain[15][34]and *loss-of-function* (LRRK^ex1^ mutant) [35–37].

Fully effective medications to treat neurodegenerative diseases are currently lacking and the discovery of novel drug targets for long-sought therapeutics is a great challenge for researchers and clinicians. The use of plant extracts is largely employed worldwide in traditional medicine, constituting the basis of health care in many societies, to treat disparate pathologies [38]. The well-known therapeutic properties of the medicinal plants have been investigated in various animal models and the observations of such investigations have served in many instances as the basis of new drugs development [39][40][33]. A common plant of the Indian flora, also found in Southern Europe, including Sardinia (Italy), is *Withania somnifera* (*Ws*) Dunal. Its roots, used in Ayurvedic medicine for many central nervous system disorders [41][42], are a valuable herbal medication and the recognized pharmacological effects of *Ws*, such as anti-oxidant, neuroprotection and functional recovery made it of prime interest also in the treatment of PD [43][44].

The aim of this paper was twofold: on one hand to confirm the validity of the LRRK^ex1^mutant [35][37], from now on named *LRRK2 WD40 loss-of-function* (LRRK2^WD40^), as animal model of parkinsonism in *Dm*; on the other hand, to investigate the antiparkinsonian potential of the standardized methanolic extract of *Wse* roots on this mutant, as compared to *Dm* wild type (WT, Canton-S). To this end we tested lifespan, climbing activity, electrophysiological muscle parameters and subcellular ultrastructure (mitochondria and lysosomes) of the neurons involved in the motor circuitry, as those present in the *Dm* thoracic ganglia.

## Materials and Methods

For these experiments we used adult wild type (WT; Canton -S) and LRRK2^WD40^mutant (LRRK^ex1^, #34750, from Bloomington Stock Center) *Drosophila melanogaster* (*Dm*) males. After emergence from pupae, WT and LRRK2 mutant males were separated. WT and mutant flies were reared on a standard cornmeal-yeast-agar medium in controlled environmental conditions (24–25°C; 60% relative humidity; light/dark = 12/12 hours). In addition, groups of mutant and WT flies were reared on a standard medium supplemented with the standardized methanolic extract of *Withania somnifera* root (*Wse*) (gift of Natural Remedies Ltd, Bangalore, India) at three different concentrations (0.1, 1 and 10% w/w) whereas other independent groups of WT and mutant flies were reared with 0.01% (0.5 mM) L-3,4-dihydroxyphenylalanine (L-Dopa). *Wse* and L-Dopa were added once the mixture was stirred for 10 min and had cooled down sufficiently[45].All treatments were performed in two combinations concerning their life cycle: as adults (L^-^/A^+^) or from larvae and adults (L^+^/A^+^). Standard genetic procedures were used during the study.

### Survival curves

With the aim of selecting the optimal *Wse’*s concentration to perform the whole study, *Dm* were grown on standard diet supplemented with different concentrations of *Wse* at 25°C. Cohorts of 60 flies (6 flies/tube) from each experimental group (i.e. *Wse*-untreated and *Wse-*treated WT, *Wse*-untreated and *Wse-*treated LRRK2^WD40^) were monitored every 2 days for their survival. Mortality was analyzed using Kaplan-Meier survival curves and the statistical comparisons were made with a Gehan-Breslow-Wilcoxon test. All experiments were done in triplicate.

### Climbing assay

The climbing assay (negative geotaxis assay) was used to assess locomotor ability [46]. Climbing data were obtained from different age groups (**I**: 3–6; **II**: 10–15; **III**: 20–25 days old) of untreated-WT, *Wse*-untreated and *Wse*-treated LRRK2^WD40^ mutants. Cohorts of 30 flies from each experimental group were subjected to the assay. Flies were placed individually in a vertically-positioned plastic tube (length 10 cm; diameter 1.5 cm) and tapped to the bottom. Climbing time (s) was recorded upon crossing a line drawn at 6 cm from the bottom. The number of flies that could climb unto, or above, this line within 10 seconds was recorded and expressed as percentage of total flies. Data were expressed as average ± standard error of the mean (SEM) from three experiment replications. Statistically significant differences (p<0.05) among WT, *Wse*-untreated and *Wse*-treated LRRK2^WD40^ were indicated. The statistical evaluation was made with a one-way analysis of variance (ANOVA) followed by LSD post-hoc test.

### Electrophysiological recordings

At the time of the experiments, flies from group **II** were anesthetized by using CO_2_ and carefully anchored to a wax support ventral side down, as previously reported [47][48]and placed underneath a stereomicroscope. In details, two tungsten stimulating electrodes, connected to a stimulator (Master 8, A.M.P.I, Jerusalem, IL) and a stimulus isolation unit (DS2A, Digitimer Ltd., Hertfordshire, UK) were placed into both eyes of the fly in order to activate the Giant Fiber System (GFS). Stimulus intensity and duration were adjusted in every single experiment until the muscle response was detected; maximal stimulation intensity was not greater than 10 V, and stimulus duration was not greater than 0.5 ms. A ground tungsten wire was placed into the fly abdomen. A borosilicate recording electrode, shaped by a puller (P97, Sutter Instruments, Novato, CA) with a resistance of 40-50MΩ when filled with 3M KCl, was placed into the right or left backside of the fly in order to record Post Synaptic Potentials (PSPs) from the Dorsal Longitudinal Muscle fibers (DLMs). PSPs were recorded with an Axopatch 2-B amplifier (Axon Instruments, Foster City, CA), filtered at 0.5 kHz and digitized at 1 kHz. PSPs were recorded in bridge mode, measured using peak and event detection software pCLAMP 8.2 (Axon Instruments, Foster City, CA) and analyzed off-line by pCLAMP fit software (Axon Instruments, Foster City, CA). All recordings were obtained from at least 10 different flies belonging to each experimental group (i.e. WT, *Wse*-untreated and *Wse-*treated LRRK2^WD40^). Experimenters were blind to the treatment.

Additional electrophysiological experiments were performed by applying a protocol consisting in a single GFS stimulation, delivered every 20 s, followed by PSPs recording. In this different set of experiments, the “frequency of following” was determined by delivering trains of 10 stimuli at frequencies of 100 Hz (with 10 ms between stimuli) or 200 Hz (with 5 ms between stimuli). Data are expressed as mean + SEM and one or two-way ANOVA followed by Tukey’s or Bonferroni’s post-hoc test (p<0.05), were used in order to determine significant differences between groups.

### Electron microscopy analysis

*Drosophilae* WT, *Wse*-untreated and *Wse-*treated at 1% (L^-^/A^+^) and 10% (L^+^/A^+^) LRRK2^WD40^ from group **II** were anesthetized with CO_2_ before brains and thoracic ganglia being rapidly dissected out and fixed in a mixture of 2% glutaraldehyde and 2% paraformaldehyde in 0.1 M cacodylate buffer. After several rinsing in the same buffer, the samples were post-fixed in 1% osmium tetroxide in 0.1 M cacodylate buffer for 2 h and stained overnight at 4°C in aqueous 0.5% uranyl acetate solution. Then the samples were washed several times in distilled water, dehydrated in a graded ethanol series and then embedded in SPURR resin. Roughly 70 nm thick sections, corresponding to portions of the thoracic ganglia and antennal lobes (ALs; homologous to olfactory bulbs in vertebrates), were cut with a Diatomediamond knife on a Leica EM UC6 ultramicrotome. (Leica Microsystems, Germany). Images were obtained with a FEI Tecnai G2 F20 (FEI Company, The Netherlands) transmission electron microscope equipped with a Shotky field emission gun operating at an acceleration voltage of 80 kV and recorded with a 2k x 2k Ultrascan Gatan CCD camera (Gatan, USA).

## Results

### Effects of *Wse* on the lifespan of LRRK2^WD40^

**Fig 1:**
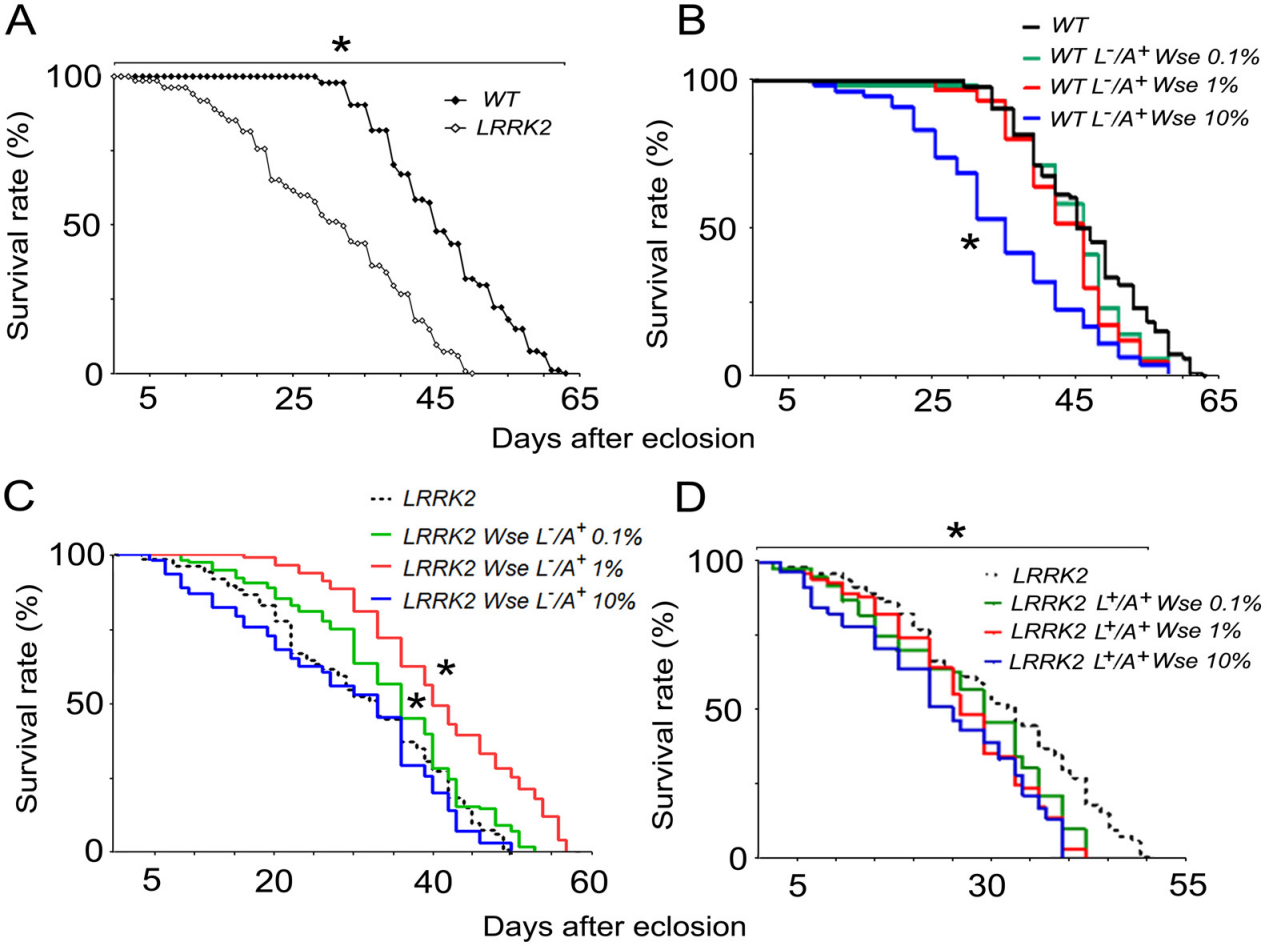
Effects of *Wse* on lifespan. (A) Lifespan, expressed as % survival rates, of wild type (WT) and LRRK2 flies. (B) Lifespan of untreated WT compared to treated WT, only when adults (L^-^/A^+^), with *Wse*, 0.1%, 1% and 10%. (C)Lifespan of untreated LRRK2 mutants compared to treated LRRK2 mutants, only when adults (L^-^/A^+^), with *Withania somnifera* extract (*Wse)*, 0.1%, 1% and 10%. (D) Lifespan of untreated LRRK2 mutants compared to treated LRRK2 mutants, from their larval stage to the end of their life-cycle (L^+^/A^+^), with *Wse*, 0.1%, 1% and 10%. *indicates p<0.05 at Kaplan-Meier survival curves (Gehan-Breslow–Wilcoxon—Graph Pad Prism 5.01), (A) untreated LRRK2 compared to untreated WT, (B) untreated WT compared to treated WT and (C-D) untreated LRRK2 compared to treated LRRK2.

Fig 1A shows that LRRK2^WD40^ mutants exhibit a significantly shorter life span than WT controls. To evaluate a possible toxic effect, *Wse* was tested at different concentrations (0.1, 1 and 10% w/w in their standard diet) as L^-^/A^+^ onto WT insects. In this respect, no significant effects were detected at any *Wse* concentration but 10% which significantly reduces the duration of life (Fig 1B) as compared to untreated WT controls. To evaluate the influence of the extract of *Wse* on the duration of life of the LRRK2^WD40^ mutants that, as reported above, demonstrated a reduced life span in respect to untreated- WT, they were treated with *Wse* at the same concentrations as L^-^/A^+^ (Fig 1C) or as L^+^/A^+^ (Fig 1D). As shown by the Kaplan-Meier survival curves, administration of *Wse* induces a statistically significant increase, even if by a different extent, in the lifespan of mutants LRRK2^WD40^, when the insects were fed in the adult stage only at 0.1% and especially 1% concentrations (p<0.05 Breslow-Gehan-Wilcoxon test). This restoring effect was lost when insects were treated at10% *Wse* L^-^/A^+^ (Fig 1C), and at any concentration when administrated to larvae and adults (L^+^/A^+^) LRRK2^WD40^(Fig 1D).The overall results are in accordance with the hypothesis that *Wse* accumulation, due to high concentration and/or long period administration, can induce a possible toxic effect.

### Effect of *Wse* on the locomotor ability of LRRK2^WD40^

**Fig 2:**
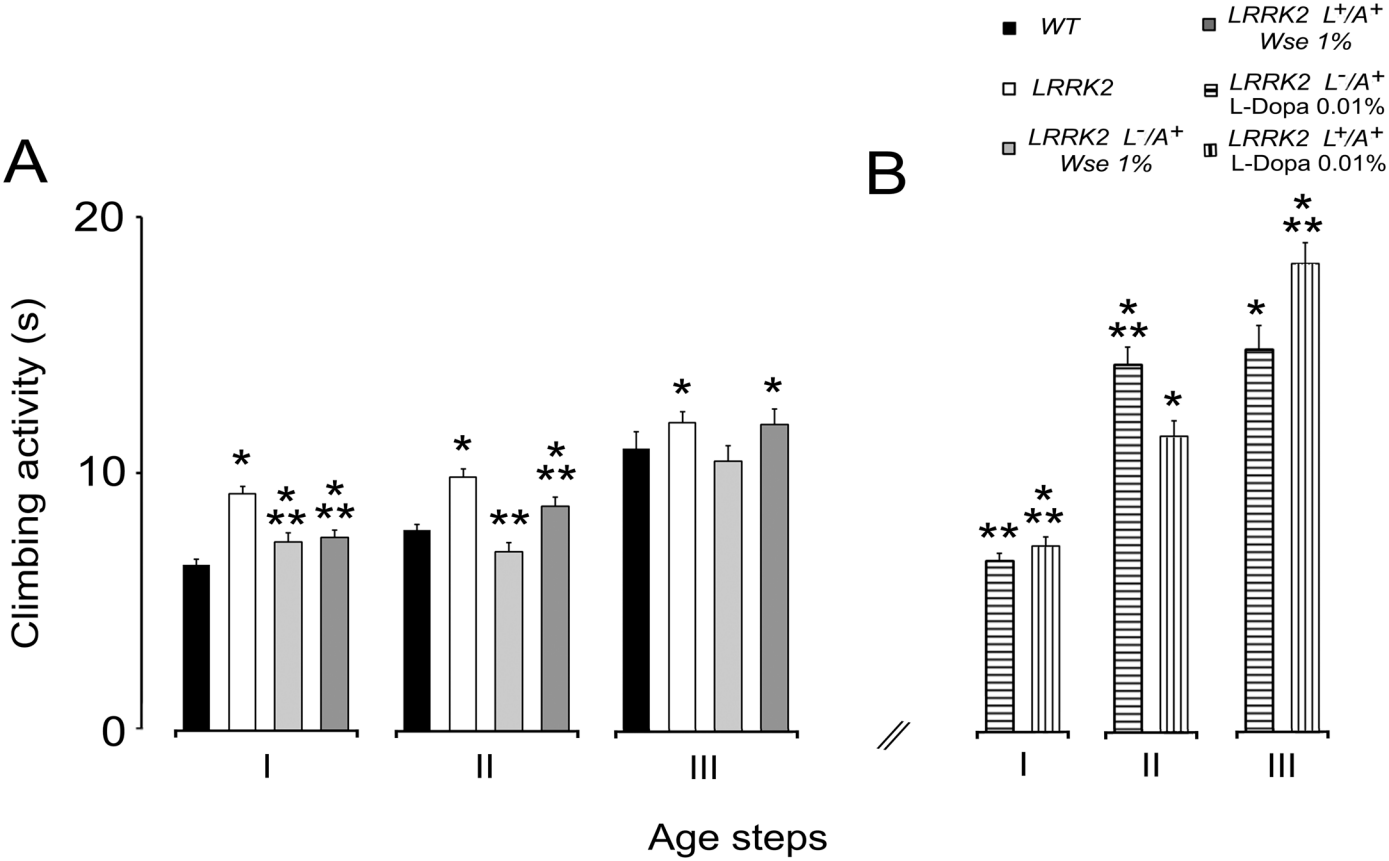
Effects of *Wse* on climbing activity. (A-B) Climbing activity of LRRK2 adult males treated with *Wse* 1% as compared with WT and untreated LRRK2 (A) and climbing activity of LRRK2 adult males treated with L-Dopa 0.01% (0.5mM) as compared with WT and untreated LRRK2 (B). Values are average ± SEM. * indicates p<0.05 at one-way ANOVA followed by LSD post hoc test as compared to WT; ** indicates p<0.05 at one-way ANOVA followed by LSD post hoc test as compared to LRRK2.

According to results obtained following *Wse* administration paralleled with life span we decided to test *Wse* at 1% w/w effects on the climbing activity (negative geotaxis) of mutants. Fig 2A shows a significant increase in the climbing time in the threeage groups tested (**I**: 3–6; **II**: 10–15; **III**: 20–25 days old) of LRRK2^WD40^ as compared to subjects of the WT group (p<0.001) with a tendency to deterioration of the motor performance with aging. The exposure of LRRK2^WD40^ to 1% w/w *Wse* as L^-^/A^+^, induces, in groups **I** and **II**, the recovery of motor disability showing a significant decrease of time to climb compared to untreated mutants; a similar result was also found in insects of groups **I-II** that were fed 1% *Wse* from larvae and adults (L^+^/A^+^). On the other hand, *Wse* administration both to L^-^/A^+^ and L^+^/A^+^ failed to significantly ameliorate motor behavior in group **III** aged flies with respect to untreated mutants. L^-^/A^+^ flies treated with *Wse* showed a clear tendency toward rescue.

Moreover, as in zebrafish LRRK2 *loss-of-function*-WD40, another PD model in which a significant rescue of motor impairment after L-Dopa treatment was obtained [49] we also tested L-Dopa at 0.01% (0.5 mM) concentration in the feeding diet of both L^-^/A^+^ and L^+^/A^+^ mutant flies. The results presented in Fig 2B show that in *Dm* mutants the administration of L-Dopa rescued the impairment of climbing activity only in insects of group **I**, while worsening the performance in groups **II-III**.

We also considered the percentages of flies that were able to complete the test and the results are shown in S1 Fig. In this respect, results confirm the rescue of insects of groups **I-II,** treated with *Wse* both as L^-^/A^+^ and L^+^/A^+^, increase with respect to untreated ones. It is noteworthy that the percentage of insects of group **II** that completed the test was 75.2% in WT, 55.6% in untreated mutants, 80.6% in L^-^/A^+^ and 69.5% in L^+^/A^+^ *Wse*-treated mutants. In group **III**, the percentage of mutant insects achieving the target was the same no matter the treatment (being 40.9%, 43.4% and 37.9%, respectively) while more than 52% of WT insects accomplished the task, according to the evaluation criterion (10 sec).

The percentages of flies that were able to complete the test after L-dopa administration are shown in S1B Fig and demonstrate that the worsening was positively correlated to age and treatment duration. Thus, the effects of *Wse* as well as those of L-Dopa administration decrease with age but that of L-Dopa was drammatic. In fact, group **III** of L-Dopa-treated flies as L^+^/A^+^ the percentage achieving the target was only 15%.

### Effects of *Wse* on the kinetic properties of evoked PSPs recorded from DLM in LRRK2^WD40^

**Fig 3:**
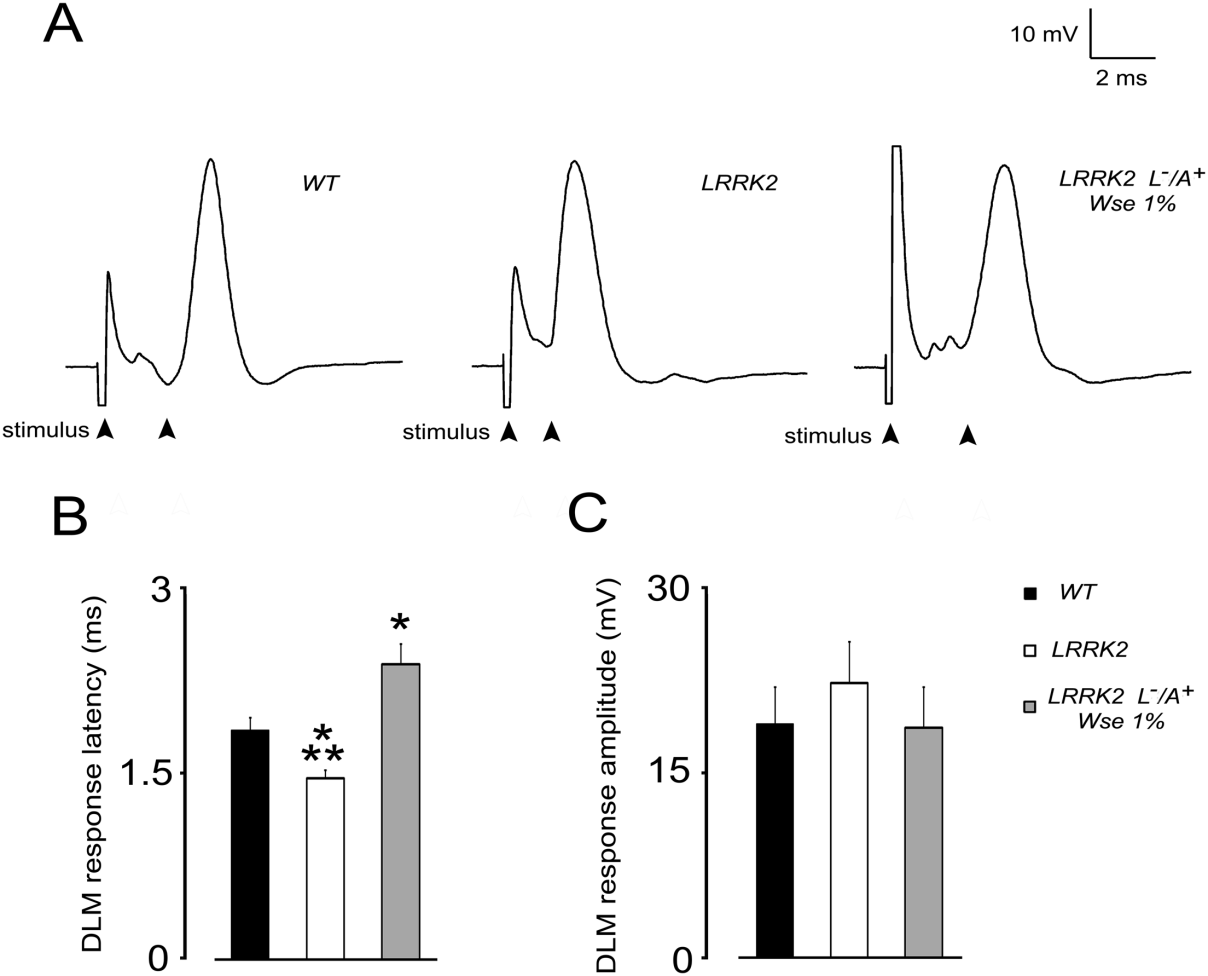
Effect of LRRK2 gene mutation and treatment with *Wse* 1% (L^-^/A^+^) on PSP latency and amplitude recorded from *Drosophila* DLM. (A) Representative traces obtained from three different flies in which PSP latency is calculated as the time (ms) from stimulus application to the peak of PSP (black arrows). (B, C) Bar graphs represent the mean ± SEM of PSP latency (ms) and amplitude (mV) recorded from flies of the indicated experimental groups. *indicates p< 0.05 compared to WT, **indicate p<0.05 compared to treated LRRK2; one-way ANOVA, followed by Bonferroni post-hoc test.

In order to detect potential changes in the function of the DLM neuromuscular junction of LRRK2^**WD40**^ flies, from group **II**, we first evaluated the basal kinetic properties of evoked PSPs (ePSPs) recorded from the DLM after GFS electrical stimulation. More precisely, we evaluated the response latency, that is the time between stimulation of the GFS and subsequent muscle PSP peak, and PSP peak amplitude, that is the maximal muscle depolarization from baseline value. Fig 3 shows that the basal properties of ePSPs recorded from DLM muscle of WT animals results in a latency of 1.84 ± 0.1 ms and in an averaged amplitude of 19 ± 3 mV. Notably, LRRK2^WD40^ mutation results in a significant decrease (21%, p< 0.05) of ePSPs latency when compared to WT animals (Fig 3A and 3B). Such effect was no longer apparent in LRRK2 (L^-^/A^+^) flies that were treated with *Wse* 1%. Surprisingly, latency in LRRK2 treated flies was significantly higher with respect to both WT as well as untreated LRRK2 animals. No significant change was detected in PSP peak amplitude among flies from the different experimental groups (Fig 3A and 3C).

### Effects of *Wse* on the PSP responses to high frequency stimulation of GFS of LRRK2^WD40^

**Fig 4:**
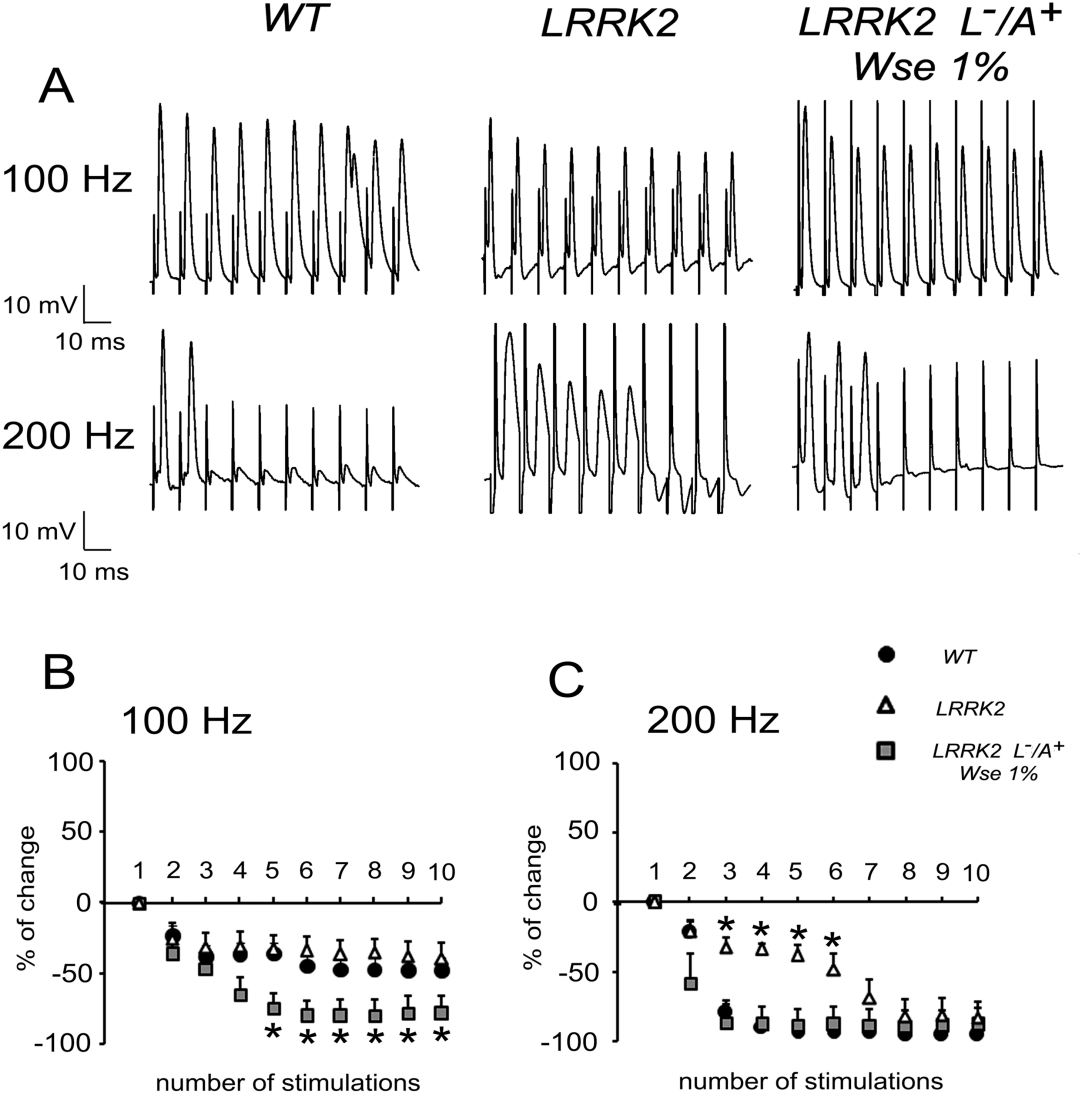
Effect of LRRK2 gene mutation and treatment with *Wse* on the “frequency of following” recorded in *Drosophila* DLM. (A) Representative traces obtained from three different flies in which PSPs were evoked in response to 10 stimulations at 100 (top) or 200 Hz (bottom). (B,C) Scatter plot graphs showing the changes in PSP amplitude following stimulation at 100 (B) or 200 Hz (C). All values are expressed as the mean ± SEM of the % relative to the amplitude of the first PSP. *indicates p< 0.05 compared to WT and *Wse*-untreated LRRK2 (B) and compared to WT and *Wse*-treated LRRK2 (C), two-way ANOVA.

We then tested flies by recording the “frequency of following” which consisted in applying a train of 10 stimuli at different frequencies (100 or 200 Hz) to GFS. As previously reported [48], in WT flies, a train of 10 stimulations at 100 Hz induced repetitive responses of DLM with minimal decrement of PSP amplitude as compared to the first PSP (Fig 4A and 4B). The response to 100 Hz stimulation in LRRK2^WD40^ was not different from that observed in WT (Fig 4A and 4B). In contrast, the response to 100 Hz in *Wse*-treated LRRK2^WD40^ (L^-^/A^+^) flies revealed a significant decrement of PSP amplitude when compared to the first PSP (Fig 4A and 4B). At the higher frequency of electrical stimulation, the DLM responses of WT started to decrease in amplitude after the 2nd PSP with 200 Hz stimulations (Fig 4A and 4C). The same protocol of recording at 200 Hz performed in untreated LRRK2^WD40^ flies showed that DLM responded to each of the 10 stimulations whose amplitude of PSPs was only slightly diminished (Fig 4A and 4C). In treated LRRK2^WD40^ (L^-^/A^+^) insects stimulations at 200 Hz elicited DLM PSPs which, similarly to WT flies, had amplitudes that decreased with respect to the first PSP. Two-ways ANOVA revealed a significant effect of the untreated-LRRK2^WD40^ group compared to WT when responding to the 200-Hz stimulation (P<0.05)

### Effects of *Wse* on the subcellular morphology of LRRK2^WD40^

**Fig 5:**
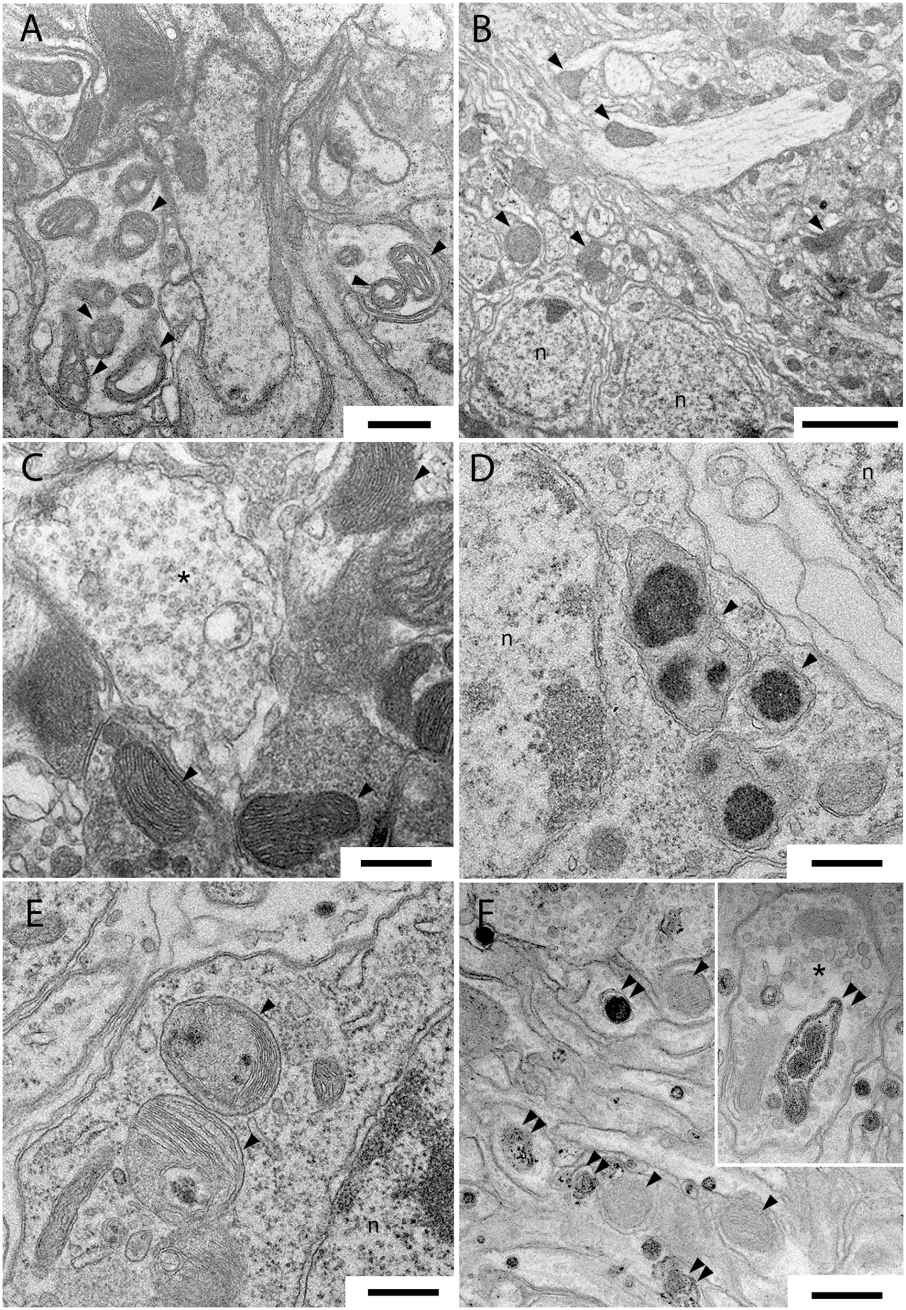
Samples of transmission electron microscopy images of thoracic ganglia and antennal lobes in *Drosophila* LRRK2 mutant (A) and after treatment with 1% in L^-^/A^+^ insects (B, C) and 10% L^+^/A^+^ (D-F) extract of *Wse*. (A) abnormal mitochondria in the thoracic ganglia neuropil of *Drosophila* LRRK2. (B, C) conventional mitochondria in thoracic ganglia of *Drosophila* LRRK2 after treatment with 1% *Wse* L^-^/A^+^imaged at low (B) and higher magnification (C). (D, E) abnormal mitochondria in *Drosophila* LRRK2 thoracic ganglia cell bodies after treatment with 10% *Wse*L^+^/A^+^. Note the irregular electron-dense substance clearly recognizable inside the mitochondria. (F and Inset) numerous endosomes are present inside the antennal lobes neurites of *Drosophila* LRRK2 after treatment with 10% *Wse*. Scale bars are 0.5 μm except in B that is 2.5 μm.

Fig 5 shows representative transmission electron microscopy images of thoracic ganglia and antennal lobes (ALs) of untreated *Dm* LRRK2 mutants (A) and of 1% and 10% *Wse*-treated, as L^-^/A^+^ (B and C) and as L^+^/A^+^ (D-F), insects. In mitochondria of the thoracic ganglia of LRRK2 mutants, we observed regions with several damaged, swollen, and with clearly fragmented cristae, that we failed to find in the corresponding regions after treatment with 1% *Wse* (in Fig 5 compare A with B and C). However, after treatment with 10% *Wse* L^+^/A^+^, we observed, in the corresponding regions of the thoracic ganglia, numerous altered mitochondria with a granular, irregularly shaped electron-dense material in their matrix (Fig 5D and 5E). Moreover after the same treatment we observed, in *Drosophila* LRRK2^WD40^ALs numerous late endosomes/ phagosomes vacuoles inside presynaptic terminals and dendrites (Fig 5F).

## Discussion and Conclusions

One of the aims of the present study was to validate the use of LRRK2^WD40^ as a model of PD. In this respect, these mutant flies show reduced lifespan, and motor impairments (face validity) and mitochondrial dysfunctions (construct validity) that characterize Parkinsonism. Furthermore, this study was aimed at evaluating the action of the standardized extract of the roots of *Withania somnifera (Wse*) and its possible neuroprotective effects on the Parkinson’s genetic model of *Drosophila melanogaster* LRRK2^WD40^. Although almost all of the mutations in LRRK2 have a number of related features, these mutants object of the present study lack, in particular, the WD40 domain responsible for coding a protein chaperone known to be involved in a number of cellular functions such as cytoskeletal, neurotransmitter vesicular pathway and lyso-endosomal activities [25] The results presented here show that the addition of 1% *Wse* to standard diet of only LRRK2^WD40^ adults (L^-^/A^+^), but not of L^**+**^/A^+^, significantly **a)** increases their lifespan compared to untreated controls and **b)** improves their locomotor abilities and **c)** affects evoked electrophysiological parameters. Furthermore, in thoracic ganglia, under electron microscopy observation, we found that *Wse* administration dramatically rescued the mutation-related loss of mitochondrial structural integrity. Interestingly, *Wse* chronic administration to flies as L^+^/A^+^, no matter the concentration, induces a worsening of symptoms associated with parkinsonism and a further decrease of lifespan as compared to WT controls as well as to untreated LRRK2^WD40^ (Fig 1B)

The flight muscle degeneration accompanied by defects in motor activity [50–52] detected in our study is probably related to dysfunction of dopaminergic neurons. Accordingly, in a zebrafish model LRRK2 *loss-of-function* in the WD40 domain, it was previously reported a rescue of motor impairment following L-Dopa administration in the early larval stage from days post fecundation (DPF) 5 to 6 [49]. Notably, although this and our model of LRRK2 *loss-of-function* differ in a number of factors such as animal species, life period and duration of L-Dopa administration, the present results also demonstrate an improvement of motor deficit (climbing activity) in the mutants of the group **I** treated as L^-^/A^+^. However, extension of the treatment to flies of group **II** and **III** did not rescue the mutation-dependent impairment but elicited a worsening in both L^-^/A^+^ and L^+^/A^+^ treated flies (Fig 2B).

The observed rescue of impaired motor ability by *Wse* administration to LRRK2^WD40^ *Dm* while confirming the condition of mutation-dependent impaired motility, as shown in tests of climbing (Fig 2), also supports the suggestion that *Wse*’s effects might be attributable to increased neurotransmission [53][54]that would result in a better locomotion. Electrophysiological data showed that mutation of the LRRK2 gene was associated with a significant decrease in PSP latency when compared to WT animals, an effect that was no longer apparent in LRRK2 (L^-^/A^+^) flies that were treated with *Wse* 1%. However, no significant change of PSP peak amplitude was detected among flies from the different experimental groups suggesting that in LRRK2^WD40^ mutants there is a higher probability of (but not necessarily an optimally coordinated) muscle contraction compared with WT without changes in muscle contraction *per se*. Surprisingly, *Wse* treatment was able to revert the effect of mutation making the response latencies recorded in LRRK2 (L^-^/A^+^) treated flies much higher as compared with both untreated LRRK2 and WT flies. The decrease in PSP latency together with the decreased responsiveness to high frequency stimulation observed in untreated-LRRK2^WD40^ flies appears to well correlate with the motility impairment observed in these flies. As for the possible mechanism, Augustin and colleagues [48] reported that recording the “frequency of following”, a GFS train stimulation at 200 Hz induced in WT a significant decrement of PSP amplitude relative to the first PSP because the intermediary synapses do not have sufficient time to recover between stimuli. Conversely, a stimulation train at 200 Hz performed in untreated LRRK2^WD40^ flies showed that, relative to the first PSP, the amplitude of PSPs was only slightly diminished, starting from the second response, and treatment with 1% *Wse* made the responses similar to those observed in WT. Thus, the effect of *Wse* on the functional changes associated with the mutation clearly discloses a beneficial aspect of this treatment. At this time, we cannot explain in deep details the abnormal effect of *Wse* treatment in LRRK2 flies (i.e. increased PSP latency and exacerbated effect on 100 Hz response vs WT), and this might at least in part be justified recalling the complexity of the projection pathway from the brain to the thoracic ganglion, where axons form electrical synapses with interneurons and the latter form chemical synapses on each motor neuron innervating the DLMs [55][56]. However, mutation of LRRK2^WD40^ may be correlated with a significant impairment in neurotransmitter release from presynaptic terminals [25][57].

The impaired motility shown by the LRRK2 mutants is paralleled by the presence of scattered abnormal mitochondria in their thoracic ganglia, an observation corroborated by other studies that suggest the involvement of LRRK2^WD40^ in mitochondrial homeostasis, responsible of mitochondrial degradation[58][59]. Intriguingly, the conventional mitochondrial morphology of LRRK2^WD40^ flies observed after treatment with 1% *Withania* extract, and paralleled by an improvement in their motor capacity, suggests that *Wse* may also act suppressing mitochondrial dysfunction, as has been recently demonstrated for a green tea-derived catechin, epigallocatechin gallate (EGCG) [59] and as well as already demonstrated in the case of the mutant PINK1^B9^ treated with the standardized seeds extract of another plant, *Mucuna pruriens* [33].

In conclusion, based on our results we can infer that the LRRK2 *loss-of-function* in the WD^40^ domain is a plausible model that recapitulates some of the essential features of Parkinsonism and that the extract of *Ws* can be usefully employed to counteract some deficits associated with this condition. However, as demonstrated by Poddighe et al., [33] after *Mucuna pruriens* administration to *Dm* PINK1^B9^ mutant model of PD, the use of a whole herbal extract requires careful assessment. In fact, the effects of *Wse* on LRRK2^*WD40*^ might also be related to age (group I *vs* III), length of exposure (L^-^/A^+^ *vs* L^+^/A^+^) and *Wse* (0.1% *vs* 1% *vs* 10%) concentrations as suggested by the observation of its effects on climbing (Fig 2A and 2C) as well as on life duration (Fig 1C and 1D). Indeed, the negative effect of 10% *Wse* both on WT (Fig 1B) and on the *loss-of-function* LRRK2^WD40^mutant indicates that one or more components of the extract, when administered chronically and at a concentration higher than optimal, may have toxic effects. This conclusion is supported by the observation that chronic administration of *Wse* to flies as L^+^/A^+^, no matter the concentration, and also at 10% to L^-^/A^+^, induces a worsening of symptoms associated with parkinsonism and a further reduction of lifespan as compared to WT controls and untreated LRRK2^WD40^. This observation also indicates that *Wse* shows a concentration threshold, below which it does not work; b) has an optimal value for its effects; but c) whose effects at higher concentrations and/or after longer exposures became toxic. As discussed above, this suggests that *Wse* exerts its effects -as a drug- following a hormesis-like dose-response curve [60] and further highlights the need to assess the proper concentration of *Wse*. In this regard, the presence of numerous large sized lysosomes observed exclusively in the ALs of *Drosophila* LRRK2^WD40^ treated with 10% *Wse*, corroborates its toxic effect, since lysosomes increases in number and size are one of the more common cause of degenerative brain disorders [61].

## Supporting Information

S1 Fig Percentages of insects able to achieve the test. (A-B) Percentages of adult males WT, LRRK2, *Wse* 1% treated LRRK2 (A) and L-Dopa 0.01% (0.5mM) treated LRRK2 (B), that could climb unto, or above, the line drawn at 6 cm from the bottom of the tube within 10 seconds.Treatments were administered to flies both only when adults (L^−^/A^+^) and from their larval stage to the end of their life-cycle (L^+^/A^+^), and their effects were assayed at three different age steps (**I**: 3–6; **II**: 10–15; **III**: 20–25 days) of flies’ life-span. Values are average ± SEM. * indicates p<0.05 at one-way ANOVA followed by LSD post hoc test as compared to WT; ** indicates p<0.05 at one-way ANOVA followed by LSD post hoc test as compared to LRRK2.
(TIF)

## Abbreviations

*Dm*: *Drosophila melanogaster*
LRRK2: leucine-rich repeat kinase 2
LRRK2^WD40^: LRRK2 *loss-of-functionin the WD40 domain*
PD: Parkinson’s disease
*Wse*: *Withania somnifera* extract
WT: wild type
L-Dopa: L-3,4-dihydroxyphenylalanine
GFS: Giant Fiber System
DLM: dorsal longitudinal muscles
