# Supplementary figures and images for: Functional and Morphological Correlates in the Drosophila LRRK2 loss-of-function Model of Parkinson’s Disease: Drug Effects of Withania somnifera (Dunal) Administration
Source: PLoS One. 2016 Jan 4;11(1):e0146140. doi: 10.1371/journal.pone.0146140 (PMC4699764; doi:10.1371/journal.pone.0146140)

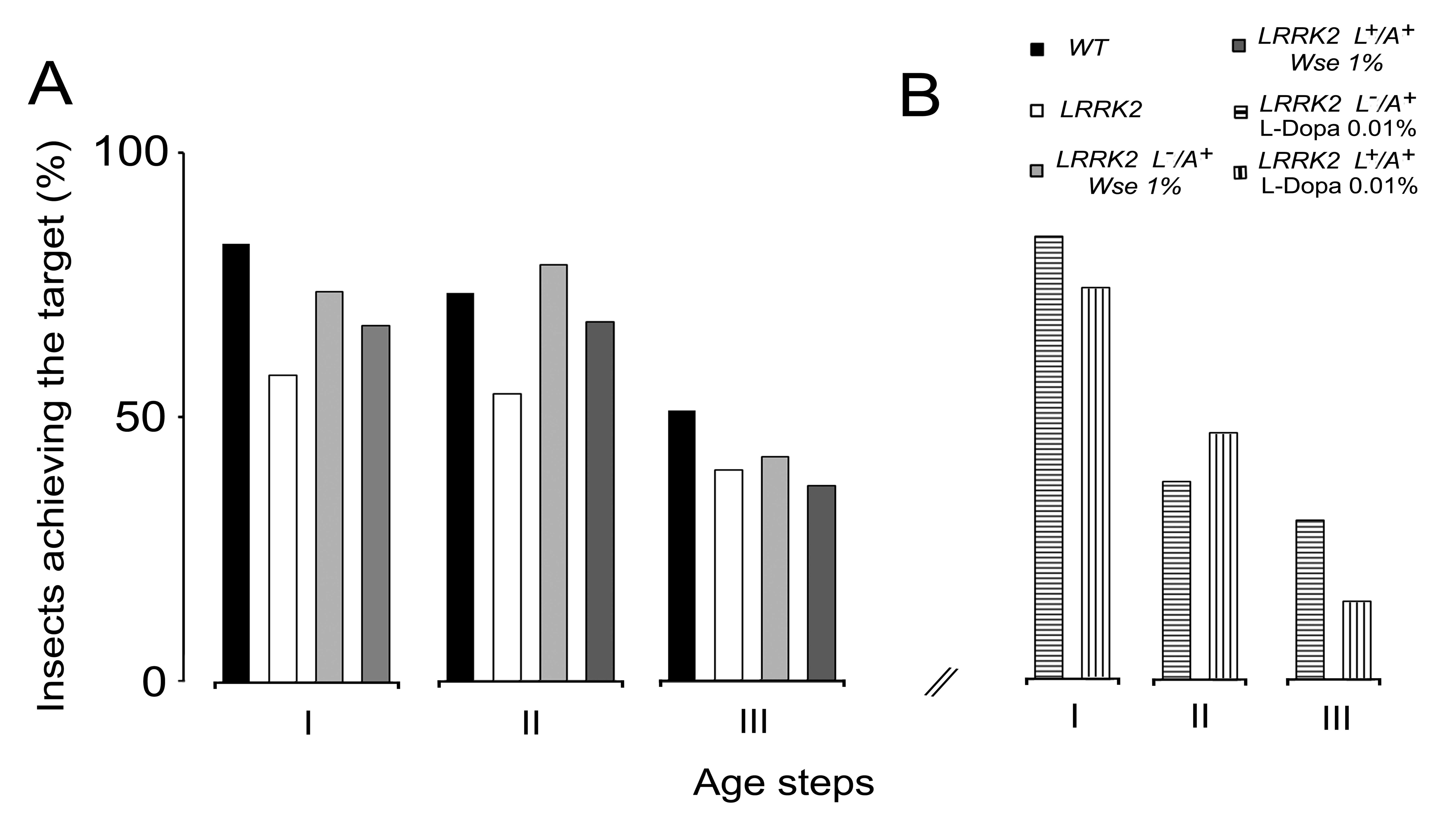

Supplement: S1 Fig — (A-B) Percentages of adult males WT, LRRK2, Wse 1% treated LRRK2 (A) and L-Dopa 0.01% (0.5mM) treated LRRK2 (B), that could climb unto, or above, the line drawn at 6 cm from the bottom of the tube within 10 seconds.Treatments were administered to flies both only when adults (L−/A+) and from their larval stage to the end of their life-cycle (L+/A+), and their effects were assayed at three different age steps (I: 3–6; II: 10–15; III: 20–25 days) of flies’ life-span. Values are average ± SEM. * indicates p<0.05 at one-way ANOVA followed by LSD post hoc test as compared to WT; ** indicates p<0.05 at one-way ANOVA followed by LSD post hoc test as compared to LRRK2. (TIF) [file pone.0146140.s001.tif]
